# HeartMate 3 implantation in a patient with mechanical mitral valve prosthesis: intentional leaflet fracture and sewing ring preservation

**DOI:** 10.1093/jscr/rjag549

**Published:** 2026-07-06

**Authors:** Denyan Mansuroglu, Ali Dogan

**Affiliations:** Department of Cardiovascular Surgery, Gaziosmanpasa Hospital, Istinye University, Faculty of Medicine, Gaziosmanpasa, Istanbul, Turkey; Department of Cardiology, Gaziosmanpasa Hospital, Istinye University, Faculty of Medicine, Gaziosmanpasa, Istanbul, Turkey

**Keywords:** left ventricular assist device, HeartMate 3, mechanical mitral valve, redo cardiac surgery, advanced heart failure, tricuspid annuloplasty

## Abstract

Management of a pre-existing mechanical mitral valve prosthesis during durable left ventricular assist device (LVAD) implantation remains controversial. Retaining the prosthesis may predispose to leaflet immobility, thrombosis, and inflow obstruction, whereas full prosthetic replacement adds substantial operative risk, particularly in redo cardiac surgery. We describe a novel surgical strategy combining removal of the mobile prosthetic components with preservation of the sewing ring during HeartMate 3 implantation. A 49-year-old woman with advanced heart failure and previous mechanical mitral valve replacement underwent HeartMate 3 implantation with concomitant tricuspid annuloplasty. The prosthetic leaflets and visible pivot components were intentionally fractured and removed, while the sewing ring was preserved in situ. At 12-month follow-up, the patient remained clinically stable without thromboembolic events, device malfunction, or inflow obstruction. This strategy may represent a feasible alternative to complete prosthetic replacement in selected redo patients.

## Introduction

Management of a pre-existing mechanical mitral valve prosthesis during durable left ventricular assist device (LVAD) implantation remains controversial. Retention of the prosthesis may predispose to leaflet immobility, thrombosis, and inflow obstruction under continuous-flow conditions, whereas complete prosthetic replacement increases operative complexity and surgical risk, particularly in redo procedures [1–3]. We describe a novel surgical strategy during HeartMate 3 implantation consisting of intentional removal of the mechanical valve leaflets and visible pivot components while preserving the prosthetic sewing ring in situ. To our knowledge, this is the first reported case using this technique.

## Case presentation

**Figure 1:**
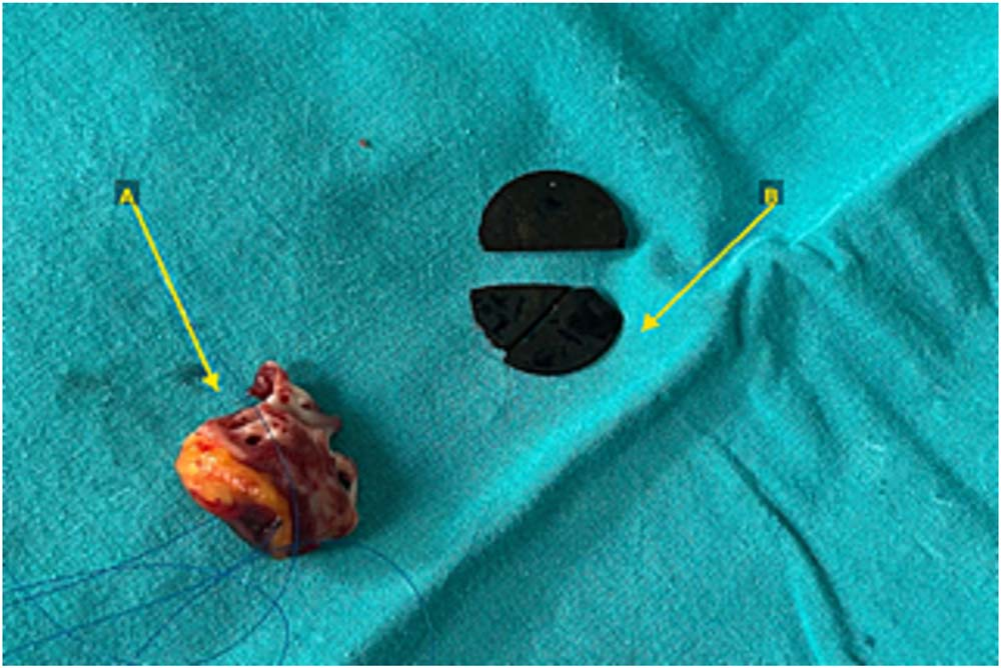
Intraoperative findings during HeartMate 3 implantation. (A) Left ventricular apical myocardial core removed during creation of the inflow cannula site. (B) Metallic leaflets and pivot/strut components extracted from the mechanical mitral prosthesis after intentional fragmentation. The prosthetic sewing ring was preserved in situ.

**Figure 2:**
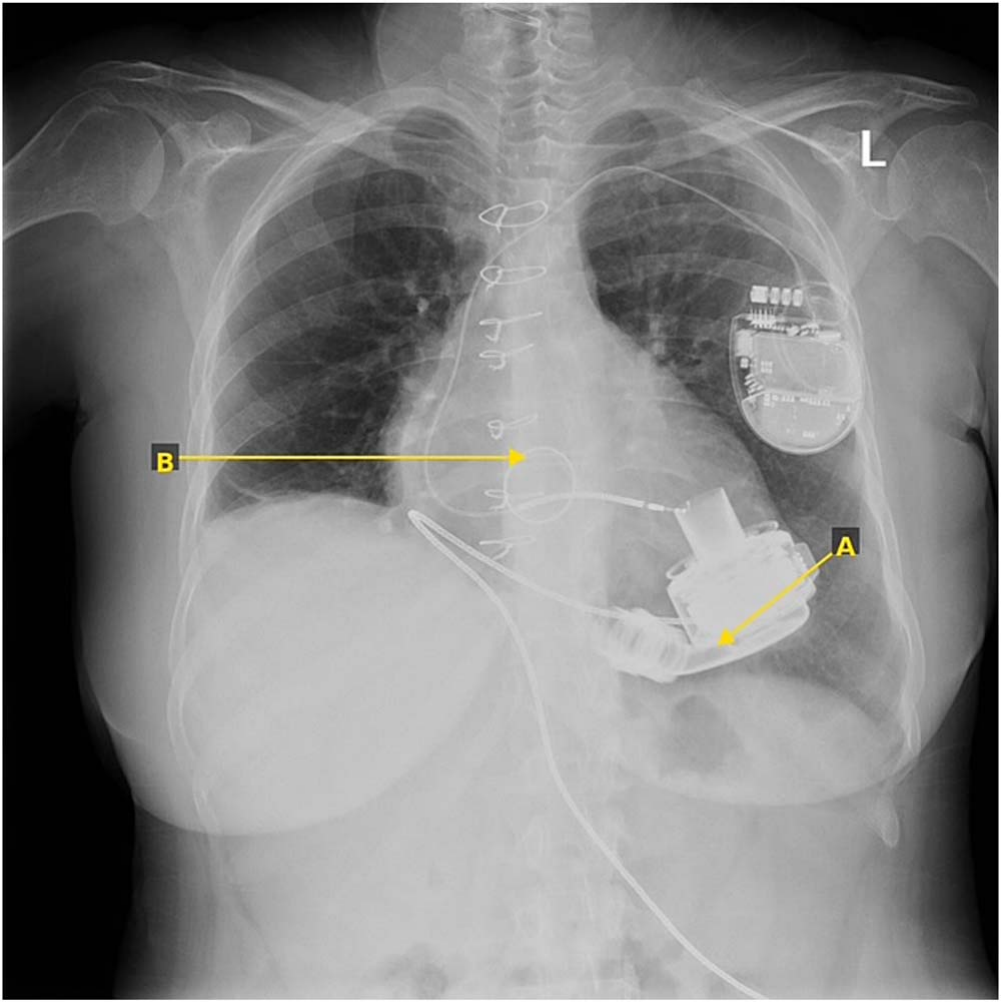
Postoperative posteroanterior chest radiograph demonstrating appropriate position of the HeartMate 3 pump, inflow cannula, and retained radiopaque mitral prosthetic sewing ring. The implantable cardioverter-defibrillator generator and transvenous leads are visible in the left pectoral region.

**Figure 3:**
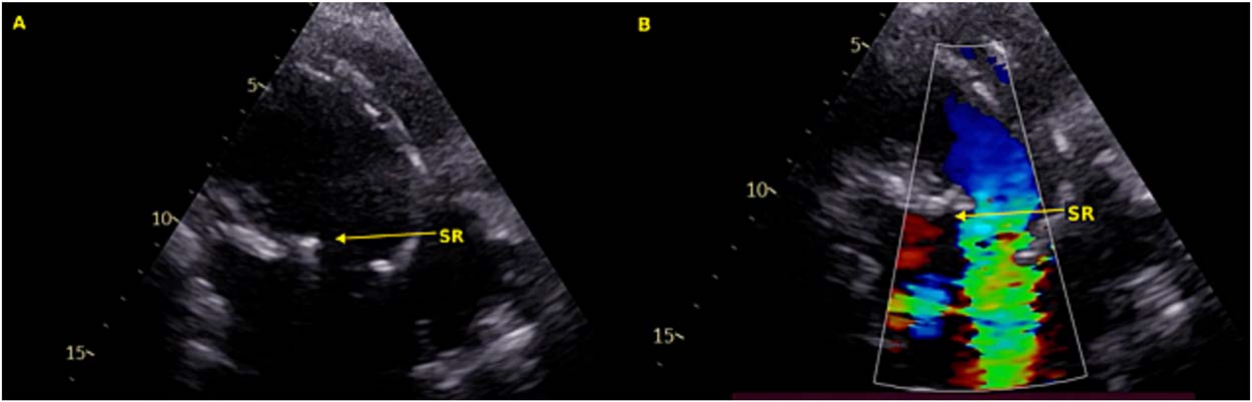
Follow-up transthoracic echocardiography (parasternal long-axis view) showing optimal apical alignment of the LVAD inflow cannula without interference from the retained mitral sewing ring. Effective left ventricular unloading with reduced cavity dimensions is evident. Mild residual tricuspid regurgitation and estimated pulmonary artery systolic pressure of 40 mmHg were also documented.

A 49-year-old woman with previous mechanical mitral valve replacement, prior percutaneous coronary intervention, and implantable cardioverter-defibrillator implantation presented with progressive exertional dyspnea, peripheral edema, and ascites despite optimal medical therapy. On admission, clinical examination demonstrated volume overload with elevated jugular venous pressure and bilateral lower-extremity edema. The patient was categorized as INTERMACS profile 3, indicating advanced heart failure requiring durable mechanical circulatory support. Transthoracic echocardiography demonstrated severe biventricular dysfunction with a left ventricular ejection fraction of 18%, severe functional tricuspid regurgitation, and preserved mobility of the mechanical mitral prosthesis. Right heart catheterization confirmed elevated filling pressures and increased risk of postoperative right ventricular failure. Following preoperative optimization with intravenous diuretics and inotropic support, the patient underwent HeartMate 3 implantation with con-comitant tricuspid annuloplasty. Through a transseptal approach, the 

mechanical mitral prosthesis was inspected intraoperatively. Because of concerns regarding leaflet immobility, thrombosis, and potential inflow obstruction after LVAD activation, the metallic leaflets and visible pivot components were intentionally fractured and removed, while the prosthetic sewing ring was preserved in situ (Fig. 1). This approach avoided complete prosthetic explantation and maintained annular integrity. The postoperative course was uneventful, and the patient was discharged on postoperative day 15 on warfarin anticoagulation. At 12-month follow-up, the patient remained in New York Heart Association functional class I–II without thromboembolic complications, pump thrombosis, or device-related rehospitalization. Chest radiography and transthoracic echocardiography confirmed satisfactory LVAD position and unobstructed inflow cannula alignment (Figs 2 and 3).

## Discussion

Management of mechanical mitral valve prostheses during LVAD implantation remains controversial [3, 4]. Retaining the prosthesis may predispose to leaflet immobility, thrombosis, and inflow obstruction under continuous-flow conditions, whereas complete valve replacement increases operative complexity and bleeding risk, particularly in redo surgery [5, 6]. In this case, selective removal of the prosthetic leaflets and pivot components while preserving the sewing ring provided an alternative strategy that avoided annular reconstruction and maintained unobstructed inflow geometry. An additional advantage of this approach was facilitation of optimal inflow cannula alignment toward the mitral inflow axis, potentially improving ventricular washout and reducing thrombosis risk [7]. Concomitant tricuspid annuloplasty was also performed because of severe tricuspid regurgitation and increased risk of postoperative right ventricular dysfunction [8]. The patient remained free from thromboembolic or device-related complications at 12-month follow-up while receiving standard warfarin anticoagulation [9]. Although limited by the single-case design and relatively short follow-up, this experience suggests that selective prosthetic component removal with preservation of the sewing ring may represent a feasible option in carefully selected patients undergoing durable LVAD implantation [10–12].

## Conclusion

A pre-existing mechanical mitral valve prosthesis should not be considered an absolute contraindication to durable LVAD implantation. In selected redo patients, removal of the prosthetic leaflets with preservation of the sewing ring may represent a feasible alternative to complete valve replacement. This strategy may optimize inflow cannula alignment while avoiding the additional morbidity of repeat mitral valve surgery. Further experience is required to confirm long-term safety and reproducibility.
